# Supplementary material for: Laser-induced vapour nanobubbles improve drug diffusion and efficiency in bacterial biofilms
Source: Nat Commun. 2018 Oct 30;9:4518. doi: 10.1038/s41467-018-06884-w (PMC6207769; doi:10.1038/s41467-018-06884-w)
Supplement: Supplementary file 1 — Supplementary Info [file 41467_2018_6884_MOESM1_ESM.pdf]

SUPPLEMENTARY INFORMATION

Laser-Induced Vapour Nanobubbles Improve Drug Diffusion and Efficiency in Bacterial Biofilms

E. Teirlinck et al.

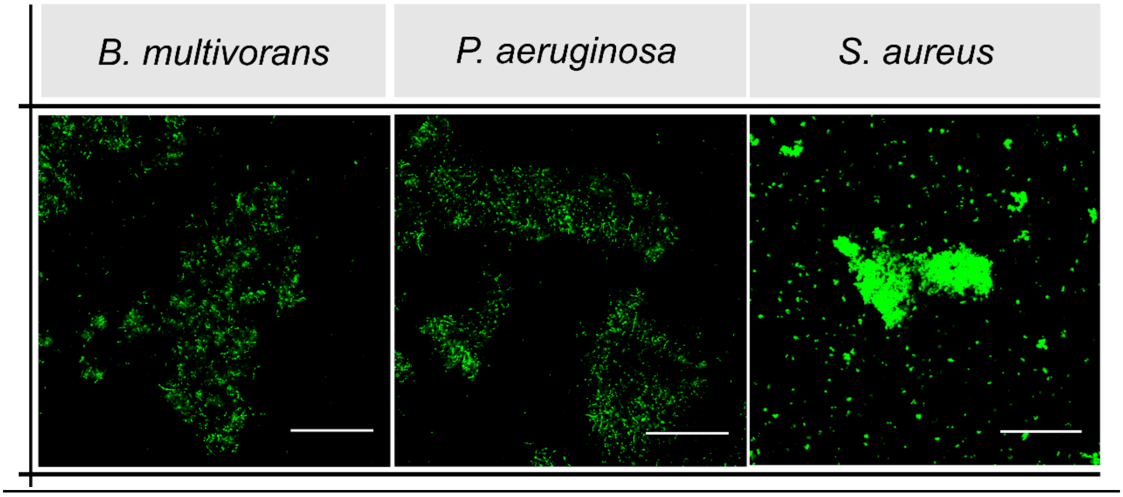

Supplementary Figure 1:

Evaluation of the biofilm structure by CLSM. Confocal image of 24-hours old biofilms of (*left) B. multivorans*, (*center) P. aeruginosa* and (*right*) *S. aureus*, grown in Mueller Hinton Broth, Lysogeny Broth and simulated wound fluid, respectively. Bacteria were stained with 20 µM SYTO59. Scale bar = 50 µm.

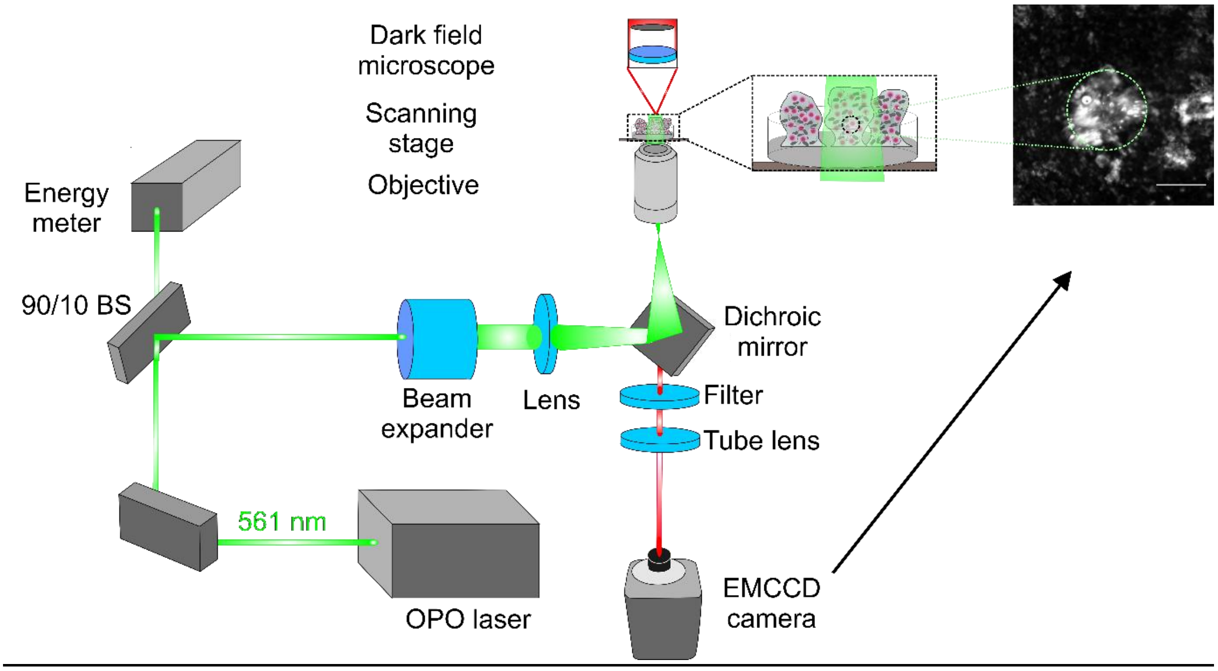

Supplementary Figure 2:
Optical set-up to generate and detect VNB.

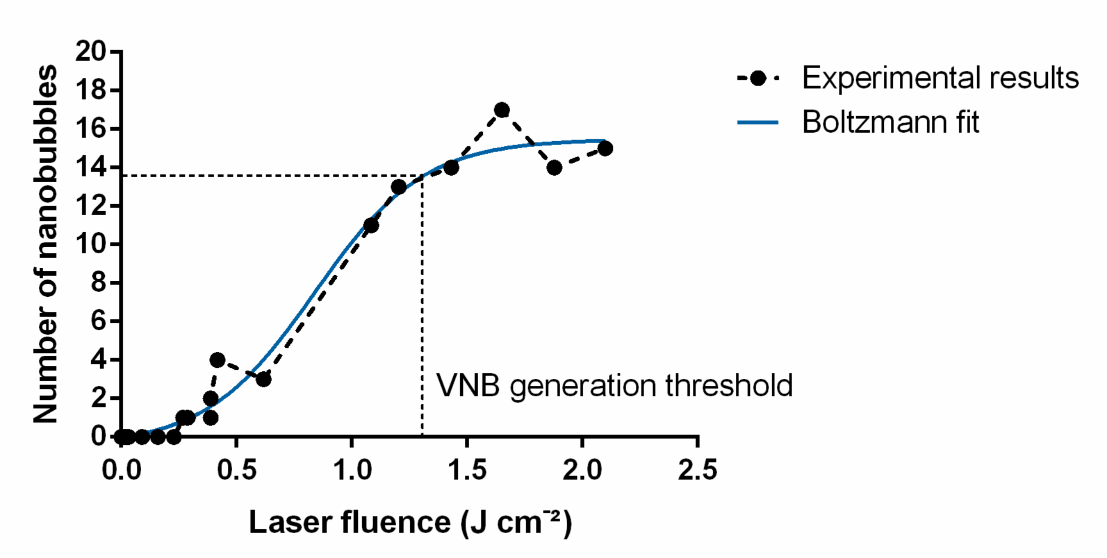

Supplementary Figure 3:
VNB generation fluence threshold of 70 nm AuNP in water. The generation threshold was defined as the laser fluence at which 90% of the AuNP in the irradiated laser beam area generated VNB.

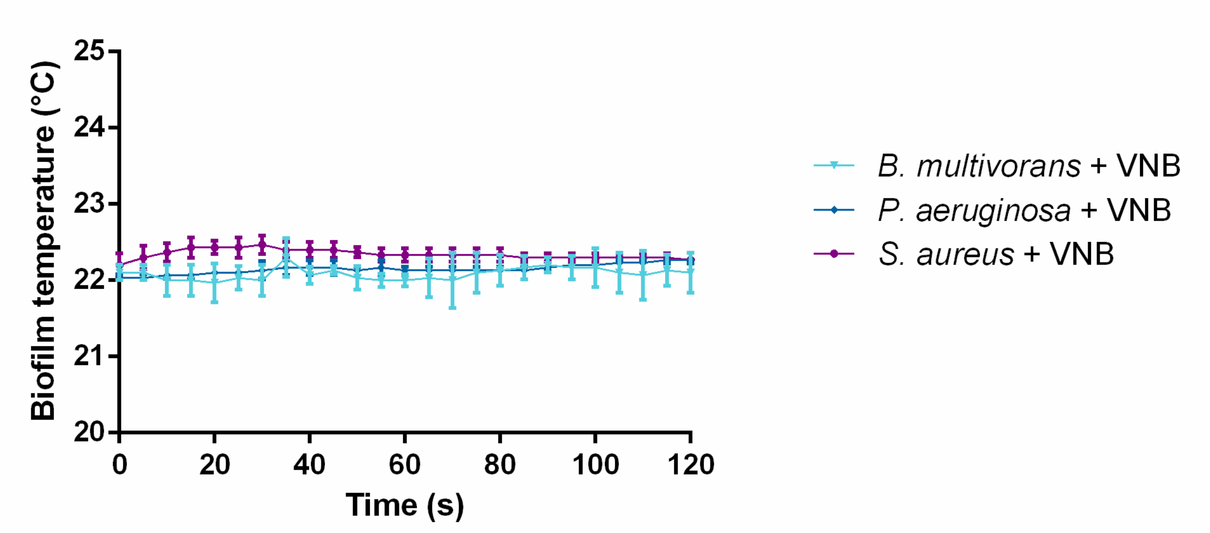

Supplementary Figure 4:

Temperature assessment during VNB-treatment. The temperature was measured with a miniature thermocouple during VNB treatment of a biofilm of *B. multivorans, P. aeruginosa* and *S. aureus* grown in a well of a 96-well titer plate (average ± SEM) *(n = 3 x 3)*.

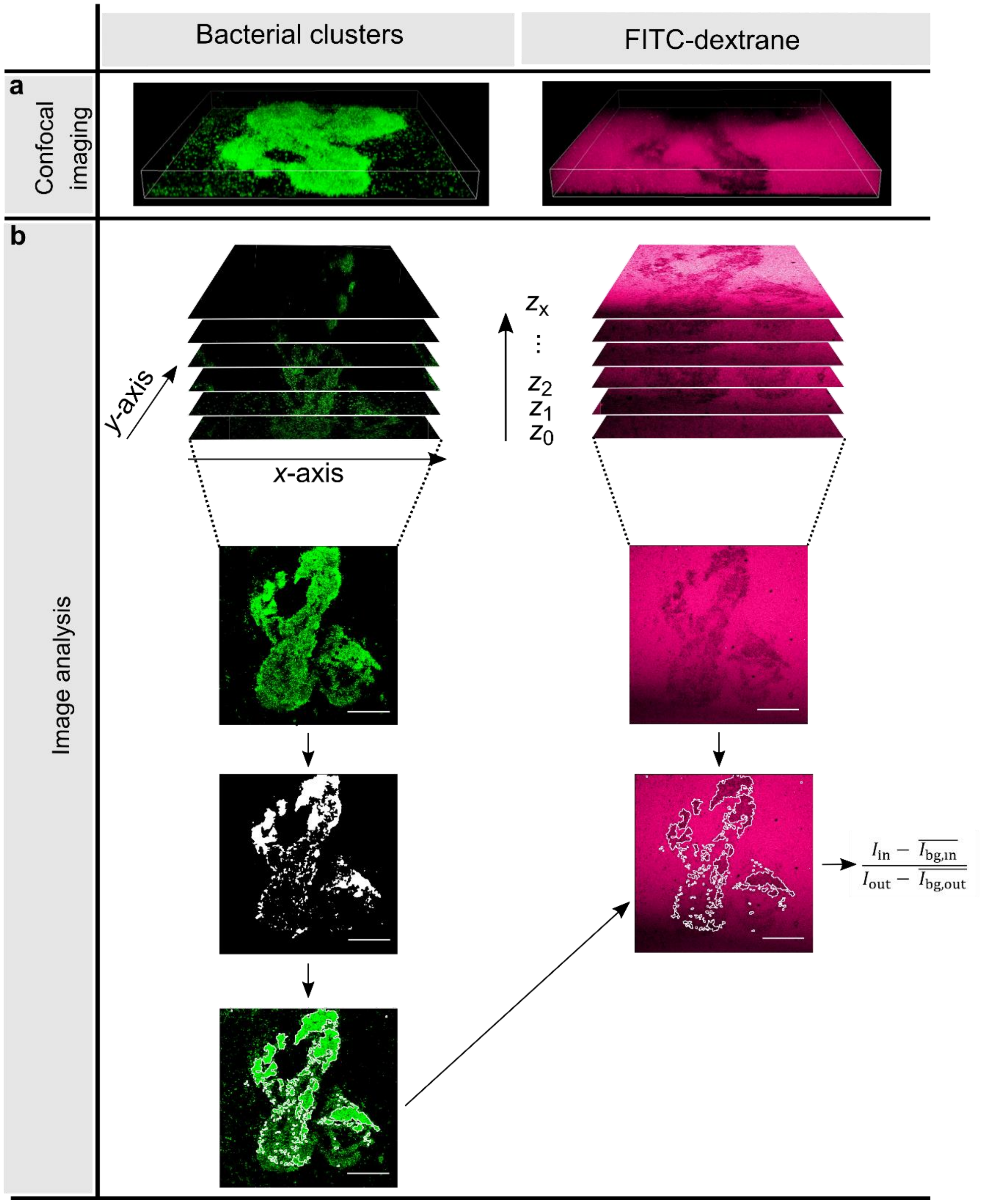

Supplementary Figure 5:
Experimental set-up to evaluate the enhanced penetration through biofilms. Bacteria are displayed in green, while FITC-dextrane is depicted in magenta.
