# Supplementary material for: Laser-induced vapour nanobubbles improve drug diffusion and efficiency in bacterial biofilms
Source: Nat Commun. 2018 Oct 30;9:4518. doi: 10.1038/s41467-018-06884-w (PMC6207769; doi:10.1038/s41467-018-06884-w)
Supplement: Supplementary file 3 — Description of Additional Supplementary Files [file 41467_2018_6884_MOESM3_ESM.pdf]

Description of Additional Supplementary Files

File Name: Supplementary Movie 1_B multivorans gold penetration.mp4
Description: Large-view 3-D confocal movies showing AuNP penetration in biofilms. AuNP were depicted in magenta, while B. multivorans, P. aeruginosa and S. aureus biofilms were depicted in green. B. multivorans: Width = 212 µm, Height = 212 µm, Depth = 35 µm; P. aeruginosa: Width = 212 µm, Height = 212 µm, Depth = 37 µm; S. aureus: Width = 212 µm, Height = 212 µm, Depth = 30 µm.

File Name: Supplementary Movie 2_P aeruginosa gold penetration.mp4
Description: Large-view 3-D confocal movies showing AuNP penetration in biofilms. AuNP were depicted in magenta, while B. multivorans, P. aeruginosa and S. aureus biofilms were depicted in green. B. multivorans: Width = 212 µm, Height = 212 µm, Depth = 35 µm; P. aeruginosa: Width = 212 µm, Height = 212 µm, Depth = 37 µm; S. aureus: Width = 212 µm, Height = 212 µm, Depth = 30 µm.

File Name: Supplementary Movie 3_S aureus gold penetration.mp4
Description: Large-view 3-D confocal movies showing AuNP penetration in biofilms. AuNP were depicted in magenta, while B. multivorans, P. aeruginosa and S. aureus biofilms were depicted in green. B. multivorans: Width = 212 µm, Height = 212 µm, Depth = 35 µm; P. aeruginosa: Width = 212 µm, Height = 212 µm, Depth = 37 µm; S. aureus: Width = 212 µm, Height = 212 µm, Depth = 30 µm.

File Name: Supplementary Movie 4_P aeruginosa after 10 pulses.mp4
Description: Dark-field imaging of repeated VNB formation inside biofilms. Gradual dispersal of biofilm clusters after applying pulsed laser treatment for 10 times.

File Name: Supplementary Movie 5_S aureus after 10 pulses.mp4
Description: Dark-field imaging of repeated VNB formation inside biofilms. Gradual dispersal of biofilm clusters after applying pulsed laser treatment for 10 times.

File Name: Supplementary Movie 6_biofilm before laser.mp4
Description: Large-view 3-D confocal movies showing FITC-dextrane penetration in biofilms. FITC-dextrane is depicted in magenta, while P. aeruginosa biofilms were depicted in green. Width = 212 µm, Height = 212 µm, Depth = 18 µm

File Name: Supplementary Movie 7_biofilm after 1 laser pulse.mp4
Description: Large-view 3-D confocal movies showing FITC-dextrane penetration in biofilms. FITC-dextrane is depicted in magenta, while P. aeruginosa biofilms were depicted in green. Width = 212 µm, Height = 212 µm, Depth = 18 µm

File Name: Supplementary Movie 8_biofilm after 2 laser pulses.mp4
Description: Large-view 3-D confocal movies showing FITC-dextrane penetration in biofilms. FITC-dextrane is depicted in magenta, while P. aeruginosa biofilms were depicted in green. Width = 212 µm, Height = 212 µm, Depth = 18 µm

File Name: Supplementary Movie 9_FITC dextrane before laser.mp4
Description: Large-view 3-D confocal movies showing FITC-dextrane penetration in biofilms. FITC-dextrane is depicted in magenta, while P. aeruginosa biofilms were depicted in green. Width = 212 µm, Height = 212 µm, Depth = 18 µm

File Name: Supplementary Movie 10_ FITC dextrane after 1 laser pulse.mp4
Description: Large-view 3-D confocal movies showing FITC-dextrane penetration in biofilms. FITC-dextrane is depicted in magenta, while P. aeruginosa biofilms were depicted in green. Width = 212 µm, Height = 212 µm, Depth = 18 µm

File Name: Supplementary Movie 11_ FITC dextrane after 2 laser pulses.mp4
Description: Large-view 3-D confocal movies showing FITC-dextrane penetration in biofilms. FITC-dextrane is depicted in magenta, while P. aeruginosa biofilms were depicted in green. Width = 212 µm, Height = 212 µm, Depth = 18 µm
